# Study on a 3D-Bioprinted Tissue Model of Self-Assembled Nanopeptide Hydrogels Combined With Adipose-Derived Mesenchymal Stem Cells

**DOI:** 10.3389/fbioe.2021.663120

**Published:** 2021-08-03

**Authors:** Guanzhou Zhou, Ailing Tian, Xin Yi, Lufeng Fan, Wenchong Shao, Han Wu, Nianfeng Sun

**Affiliations:** ^1^ Department of General Surgery, Qilu Hospital of Shandong University, Jinan, China; ^2^ Department of General Surgery, Zibo Central Hospital, Zibo, China

**Keywords:** self-assembled nanopeptides, hydrogels, adipose-derived mesenchymal stem cells, 3D bioprinting, tissue engineering

## Abstract

**Objective:** This study aimed to observe the cell growth status and multidirectional differentiation ability in a 3D-bioprinted tissue model of self-assembled nanopeptides and human adipose-derived mesenchymal stem cells (Ad-MSCs).

**Methods:** Primary Ad-MSCs were isolated, cultured, and identified by flow cytometry. Tissue models were printed *via* 3D bioprinting technology using a “biological ink” consisting of a mixed solution of self-assembled nanopeptides and Ad-MSCs. Ad-MSCs were induced into osteogenic, adipogenic, and endothelial differentiation and compared with the control groups by staining.

**Results:** The nanopeptide fiber was 10–30 nm in diameter and 200–500 nm in length under the atomic-force microscope. It had the characteristics of nano-scale materials. Flow cytometry showed that the isolated and cultured cells were positive for CD29 (98.51%), CD90 (97.87%), and CD166 (98.32%) but did not express CD31 (1.58%), CD34 (2.42%), CD45 (2.95%), or human leukocyte antigen (HLA)-DR (0.53%), consistent with the immunophenotype of Ad-MSCs. Then, a tissue model was printed using the biological ink, followed by induction of differentiation of Ad-MSCs within the tissue model. Alizarin red S staining showed the formation of calcium nodules in the osteogenesis induction experimental group, and oil red O stained lipid droplets in Ad-MSCs in the adipogenesis induction experimental group, whereas the two control groups were not stained.

**Conclusion:** Ad-MSCs from primary cultures have the characteristics of stem cells. Self-assembled nanopeptide hydrogel is a good tissue engineering material that can serve as an extracellular matrix. Ad-MSCs in the 3D-printed tissue model using a biological ink consisting of a mixed solution of self-assembled nanopeptides and Ad-MSCs grew well and still had strong differentiation ability.

## Introduction

Stem cell differentiation is not only related to the pre-existing programming inside the cell but also regulated by the microenvironment in which the cell is located. Regulation of stem cell differentiation mainly occurs between cells and between the cell and the extracellular matrix, with cytokines fulfilling the functions of signal transduction and nutrition between cells and between the cell and the extracellular matrix. Therefore, factors affecting targeted endothelial differentiation of stem cells consist mainly of three aspects, cytokines, extracellular matrix, and microenvironment (Chen et al., 2014; Rosa et al., 2019). Zuk et al. isolated, cultured, and identified adipose tissue–derived mesenchymal stem cells (Ad-MSCs) and showed that under specific induction conditions, Ad-MSCs could differentiate into osteoblasts, chondrocytes, adipocytes, cardiac muscle cells, and endothelial cells, making them new seed cells of great interest (Shojaei et al., 2013; Minonzio et al., 2014).

As we all know, extracellular matrix not only supports and connects cells, but also plays an important role in cell growth, proliferation, differentiation, intercellular signal transduction and other activities. Therefore, extracellular matrix plays an important role in the human body. The results show that the scaffold materials which can maintain extracellular matrix should have good biocompatibility, strong plasticity, certain tensile strength and no immunogenicity (Wisser and Steffes, 2003; Saracino et al., 2013; Tsintou et al., 2015). At present, the common materials for the preparation of extracellular matrix scaffold include alginate, polysaccharide and collagen, and artificial degradable materials such as PLA, polyhydroxyacetic acid and PLGA (Romagnoli et al., 1990; Saito et al., 2005). However, both kinds of biomaterials have no biological activity, can not simulate the micro environment of normal tissue, and have certain limitations, which make seed cells unable to fully play their cell activities and functions. It is difficult to use these biomaterials to study completely controllable in clinical (Diduch et al., 2000; Jiang et al., 2014).

Therefore, if the nano biomaterials can be synthesized by polypeptide molecules with well known components, the disadvantages of the two materials can be avoided and will become ideal scaffold materials for tissue engineering. In fact, it has become a hot research field in tissue engineering to obtain novel nano frame materials based on the natural self-assembly of polypeptide molecules. This kind of nano peptide frame material has better biocompatibility and degradability. The more outstanding advantage is that it has biological activity, and can easily compound different bioactive molecules according to different demands, and endows the scaffold material with “biological intelligence” characteristics (Liu et al., 2012). At present, many world-famous research groups have begun to study the biological nano scaffold materials based on polypeptide, and have achieved many gratifying results in their respective studies.

The Zhang Shuguang team discovered and extracted functional self assembling nanopolypeptides, which are an injectable polypeptide hydrogel scaffold made of natural amino acids. This kind of nano polypeptide has good biocompatibility, in which seed cells can exercise their biological activity, and has no toxic side effects (Zhang et al., 1993; Altman et al., 2000; Yokoi et al., 2005). The products can be absorbed and degraded by the body, which enables researchers to better imitate and restore the human microenvironment, and meet the nutrient, waste exchange and signal molecule exchange needed for cell growth, differentiation and proliferation (Vauthey et al., 2002; Lomander et al., 2005; Mershin et al., 2005). Many studies have shown that self assembling nano peptide hydrogels have great advantages in medical research (Drury and Mooney, 2003; Yuan and Liang, 2013a; Liangliang, 2013): 1) self-assembled nano peptides can be produced in large quantities under the artificial conditions of natural amino acids. 2) The nanopeptides composed of natural amino acids have good controllability to avoid the interference of unknown factors.3) The nano polypeptide has good biocompatibility, degradability and non-toxic side effects.4) in the C segment of the nano polypeptide, different functional peptide fragments can be combined to construct a new functional self-assembled nano peptide hydrogel biological scaffold material; 5) The self-assembled nano peptide can be degraded into natural amino acids *in vivo* and absorbed by human body.

Ion Complementary self-assembled Polypeptide RADA16-I is a self-assembled polypeptide material with folding, which has both hydrophobic and hydrophilic groups, and is called amphiphilic polypeptide. Its biggest characteristic is that the liquid polypeptide solution can be “self-assembled” into a solid hydrogel when it meets certain inducing factors such as pH change, salt ion contact. The GEL is rich in water content, and the inner pores are uniform. The size of the pores is close to that of most cells. It can be used for three-dimensional cell culture, so it can better imitate the human microenvironment, nutrients, waste exchange, and signal molecule exchange required for cell growth, differentiation, and proliferation (Zhang et al., 2005; Yuan and Liang, 2013a). The KLT sequence is a similar peptide fragment of vascular growth factor (VEGF) , which can be integrated into the c-terminal of Rada16-i and can stimulate the VEGF receptor. RGD sequence is a polypeptide of cell adhesion growth factor, which exists widely in many kinds of extracellular matrix proteins, such as adhesion protein, and is a key binding site of cell adhesion, which can promote cell adhesion growth, is a preferred sequence involving proteins that bind to the cell surface (Hersel et al., 2003).

Based on the different functions of the above three peptides, the self-assembled nano-peptide Hydrogel composed of RADA16-I, KLT and RGD can achieve different functions in tissue engineering, compared with the existing common peptide hydrogels, it has great advantages. Its raw material is simple, is through the amino acid synthesis method assembly to the polypeptide, may design the polypeptide sequence artificially, thus realizes the different function, and carries on the large-scale production; because the self-assembly polypeptide is by the simple amino acid composition polypeptide, there is no immune reaction and toxic side effects, the Biocompatibility is good; its degradation products are amino acids, non-toxic, can participate in the body’s metabolism as nutrients. These advantages make RADA16-I, KLT and RGD self-assembled nano-peptide hydrogels become excellent tissue engineering framework materials.

In recent years, with the development of stem cell technology and 3D bioprinting technology, people have begun to explore the generation of new tissue-engineered organs through 3D bioprinting (Moroni et al., 2018a; Moroni et al., 2018b). At present, some scientific research institutes have taken the lead in achieving 3D printing using biological materials and cells under sterile conditions. The printed human living cells have a survival rate of up to 90%. A few human tissues, such as ear cartilage tissue, liver units, and renal units, have been successfully printed (Byambaa et al., 2017; Choi et al., 2019; Wang et al., 2019).

We cultured and validated Ad-MSCs, prepared functionalized self-assembled nanopeptide hydrogels, utilized a mixed solution of Ad-MSCs and self-assembled nanopeptides as the “biological ink,” and printed the tissue model using 3D bioprinting technology, thereby inducing the Ad-MSCs to undergo osteogenic and adipogenic differentiation. Lastly, the success of the model was validated.

## Materials and Methods

### Isolation, Culture, and Immunophenotypic Identification of Adipose Tissue–Derived Mesenchymal Stem Cells

#### Isolation, Culture, and Passaging of Adipose Tissue–Derived Mesenchymal Stem Cells

All patients signed an informed consent form, which was approved by the hospital ethics committee. Fresh adipose tissue was cut into pieces and added to type I collagenase. After digestion, centrifugation, resuspension, and filtration, the obtained cells were added to Dulbecco’s modified Eagle’s medium (DMEM)/F12 complete culture medium, and the resulting P0 culture was placed in a 37°C, 5% CO_2_ incubator with saturated humidity. Cell growth and cell morphology were observed daily under an inverted phase-contrast microscope. The P0 Ad-MSCs were digested with trypsin and ethylene diamine tetraacetic acid (EDTA). The digestion was stopped when the cells became oval-shaped and the intercellular space became larger. The adherent cells were suspended, added again to DMEM/F12 complete culture medium, and inoculated into a new culture bottle at a 1:3 ratio with 10% fetal bovine serum (FBS) complete culture medium, which became the P1 culture. Cell morphology and growth were observed under an inverted phase-contrast microscope. Cells were subsequently passaged using the same method.

### Immunophenotyping of Adipose Tissue–Derived Mesenchymal Stem Cells

A sufficient amount of digested P3 suspension was taken and centrifuged at 1,000 r/min. The cells were resuspend with 900 µl 1 × phosphate-buffered saline (PBS) and distributed evenly into nine 2-ml Eppendorf tubes, to which PE mouse IgG1 20 μl, PE CD29 20 μl, PE CD31 20 μl, PE CD34 20 μl, PE CD45 20 μl, PE CD90 10 μl, PE CD166 20 μl, PE IgG2a 20 μl, and HLA-DR 20 µl were added, respectively. The Eppendorf tubes with added antibodies were incubated in the dark for 20 min with gentle shaking every few minutes. Cells were resuspended with PBS and then centrifuged, and this step was repeated twice to wash away the residual antibodies. The cells were finally resuspended with 500 μl 1× PBS and placed in a flow cytometry–specific tube for detection on the machine.

## Preparation and Detection of Self-Assembled Nanopeptide Hydrogel

### Preparation of Self-Assembled Nanopeptide Solution

The powders of three peptides, RADA16-I, RGD, and KLT, were dissolved with biological-grade, sterile, deionized water. Their concentrations were adjusted to 10 g/L, and the mixtures underwent ultrasonic vibration for 15 min with an ultrasonic cell pulverizer. RADA16-I, RGD, and KLT were mixed at a volume ratio of 2:1:1, and the mixture was again mixed well by ultrasonic vibration with an ultrasonic cell pulverizer to successfully prepare a self-assembled nanopeptide mixed solution. The self-assembled nanopeptide solution was diluted to a concentration of 0.01% and was ultrasonically vibrated with an ultrasonic cell pulverizer. A small amount of this 0.01% self-assembled nanopeptide mixed solution was added dropwise onto a special mica sheet for observation of the nanopeptide fiber structure by atomic-force microscopy could.

### Preparation of Self-Assembled Nanopeptide Hydrogel

The required Transwell chambers were placed in a 24-well plate and kept at room temperature overnight to allow the PBS in the 24-well plate to fully penetrate the Transwell basement membrane. The PBS solution was removed by aspiration, and the self-assembled nanopeptide mixture solution was slowly added along the edge of the Transwell chamber to replace the PBS in the 24-well plate until the surface of the PBS solution was higher than the height of the self-assembled nano-peptide mixture in the Transwell chamber. The self-assembled nano-peptide hydrogel gradually formed after standing still. The Transwell chamber was then taken out, the lower membrane of Transwell was removed, and the formed nanopeptide hydrogels were observed.

### Scanning Electron Microscope Detection of Self-Assembled Nanopeptide Hydrogel

After following the above steps to obtain self-assembled nanopeptide hydrogel, the self-assembled nanopeptide hydrogel was fixed with glutaraldehyde fixing solution and then dehydrated. The dehydrated specimens were made into electron microscope specimens, and the internal structure of the specimens was observed under SEM.

## Compound Experiments of Self-Assembled Nanopeptide Hydrogels and Adipose Tissue–Derived Mesenchymal Stem Cells

The P3 Ad-MSCs were taken, and after PBS washing, cells were added to 1 ml 0.125% trypsin-EDTA for digestion in a 37°C incubator for 5 min. The flask was gently shaken, and cells were repeatedly observed under an inverted phase-contrast microscope. When the cells became spherical and the intercellular space became large, the digestion was stopped by the addition of 1 ml of DMEM/F12 complete culture medium (10% FBS), and the adherent cells were suspended by repeated pipetting to form a single cell suspension. The suspension was added to a sterile centrifuge tube and centrifuged at 1,000 r/min after balancing. Cells were resuspended with 10 g/L self-assembled nanopeptide mixed solution and mixed by pipetting. The self-assembled nano-peptide hydrogel containing cells was placed into Transwell chambers, PBS was added to the 24-well plate, and DMEM/F12 complete culture medium (10% FBS) was added to the chamber immediately after gelation. Cell morphology and growth status were observed daily under an inverted phase-contrast microscope.

## Self-Assembled Nanopeptide Hydrogel Combined With Adipose Tissue–Derived Mesenchymal Stem Cellsas the Biological Ink for 3D-Printed Tissue Models and Induced Differentiation Assay of Cells

### Self-Assembled Nanopeptide Hydrogel Combined With Adipose Tissue–Derived Mesenchymal Stem Cells as the Biological Ink for 3D-Printed Tissue Models

Ad-MSCs of P2 were added to 0.125% trypsin-EDTA, digested in a 37°C incubator, and resuspended by repeated pipetting to form a single-cell suspension. The cells were then resuspended with the 10 g/L self-assembled nanopeptide mixed solution and mixed well. The self-assembled nanopeptide solution containing Ad-MSCs was quickly added to the sterile “cartridge” of the 3D bioprinter, and the required model parameters were adjusted using the AnyPrint software on a computer to form a cylinder with a diameter of approximately 1 cm and height of approximately 5 mm, containing small cavities of different sizes. After the parameters were set, printing was started, and the substrate used for printing was 1 × PBS. The printed 3D tissue model was quickly transferred to a six-well plate, added to DMEM/F12 complete culture medium (10% FBS), and placed in a 37°C, 5% CO_2_ cell incubator with saturated humidity. The morphology of the 3D tissue model was observed 24 h later, and the state of the cells inside the model was observed under the inverted phase-contrast microscope.

### Phalloidin Staining of Cells Inside the 3D-Printed Tissue Model

The printed tissue model was stained with phalloidin to observe the cell structure in the tissue model. After the printed tissue model was cultured for 7 days, the tissue model was washed with PBS, then sliced into paraffin sections. The sections were soaked in 0.1% Triton X-100 for 3–5 min and washed with PBS, and the cells were incubated with 1× PBS containing 1% BSA for 20–30 min. The staining solution was added dropwise to the cover glass, and the sections were stained at room temperature for 20 min, dried, sealed, observed under a laser confocal microscope, and stored in a dark environment.

### Induction of Osteogenic Differentiation of Cells in the 3D-Printed Tissue Model and Alizarin Red S Staining

The printed tissue model was cultured for 3 days, and the mesenchymal stem cell osteogenic differentiation complete culture medium (The osteogenic inducer is prepared on a complete medium with the addition of L-glutamine, dexamethasone,-sodium glycerophosphate, and vitamin C) was added to the six-well plate, followed by medium changes once every 3 days. After 4 weeks of induction, the tissue model was paraffin-sectioned and fixed with 4% paraformaldehyde. Alizarin red S staining solution was added dropwise, and the culture plate was placed under a microscope to observe the effect of osteogenic staining.

### Induction of Adipogenic Differentiation of Cells in the 3D-Printed Tissue Model and Oil Red O Staining

The printed tissue model was cultured for 3 days, and mesenchymal stem cell adipogenic differentiation medium A (fluid a is prepared by addition of L-glutamine, insulin, 3-isobutyl-1-methylxanthine, dexamethasone, Indomethacin, etc., on the basis of the Basal Medium) was added to the six-well plate. Every 3 days, this medium was alternated with adipose-derived mesenchymal stem cell adipogenic differentiation medium B (solution B is prepared by adding L-glutamine, insulin and other components on the basis of the basic medium). After five switches between media A and B (approximately 20 days), cells were kept in medium B for 7 days, until the lipid droplets became large and round. After the adipogenic differentiation induction was completed, the tissue model was frozen-sectioned. Oil red O dye working solution was added dropwise to it, and the culture plate was placed under a microscope to observe the effect of adipogenic staining.

### Induction of Endothelial Differentiation of Cells in the 3D-Printed Tissue Model and Flow Cytometry Validation

The printed tissue model was cultured for 3 days, the cells were resuspended in endothelial cell support solution, that is, 100% EGM2-MV induction culture medium, and placed into the six-well plate. The morphological changes of the cells were observed with an inverted phase-contrast microscope every day. The medium was refreshed every 3 days, and the growth of the cells was closely observed. After 4 weeks, CD31-PE was used to label cells that had gone through induction of differentiation and those that had not undergone differentiation, and immunophenotyping was performed using flow cytometry.

## Results

### Isolation, Culture, and Identification of Adipose Tissue–Derived Mesenchymal Stem Cells

#### Morphological Characteristics of Adipose Tissue–Derived Mesenchymal Stem Cells

**FIGURE 1:**
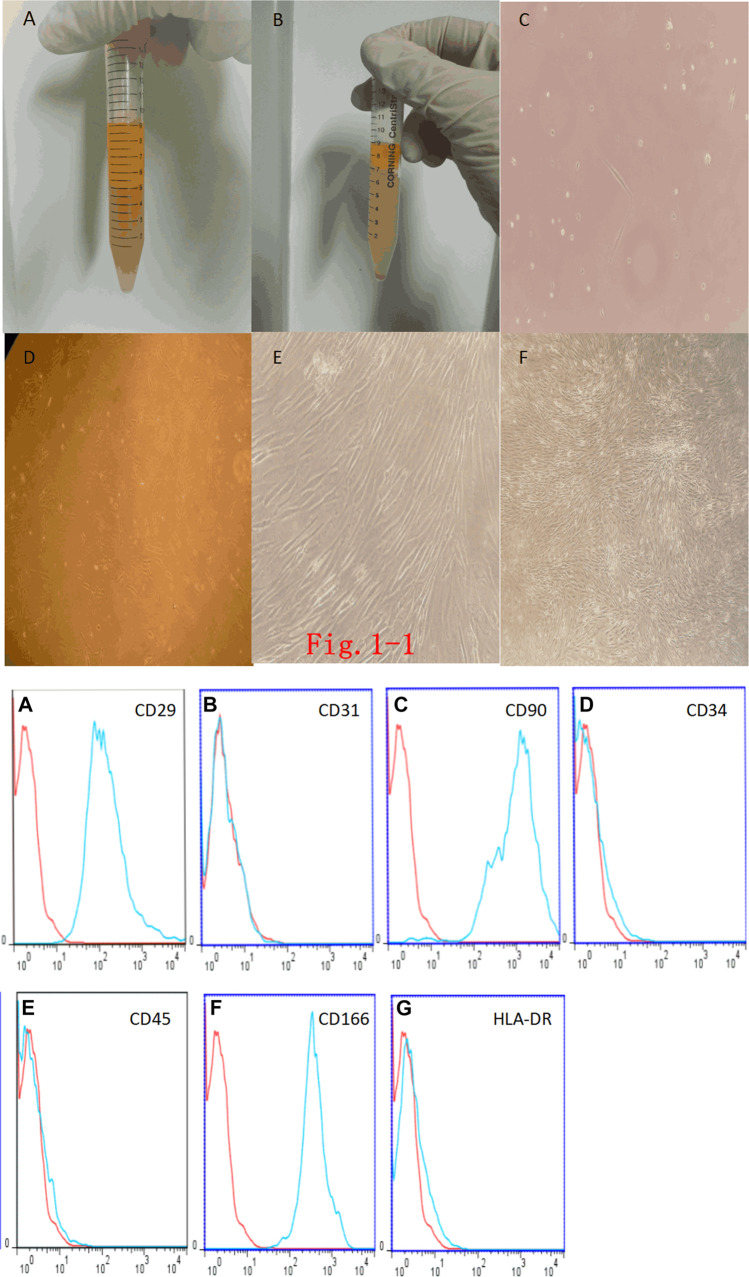
**(i)**. Fat digestion and morphology of Adipose Tissue–Derived Mesenchymal Stem Cells (Ad-MSCs). **(A)**: Fat mixed with type I collagenase. **(B)**: Complete digestion of fat by type I collagenase and centrifugation. **(C)**: Primary Ad-MSCs at 48 h (× 200). **(D)**: Ad-MSCs at 5 days (× 100). **(E)**: Ad-MSCs at 15 days (× 100). **(F)**: P2-generation Ad-MSCs (× 40). **(ii)**. Ad-MSC immunophenotyping.**(A)**: CD29-positive expression. **(B)**: CD31-negative expression. **(C)**: CD90-positive expression. **(D)**: CD34-negative expression. **(E)**: CD45-negative expression. **(F)**: CD166-positive expression. **(G)**: HLA-DR-negative expression.

Adipose tissue was diced and digested with type I collagenase. When the adipose tissue was mixed with collagenase, it mostly floated on top of the collagenase (Figure 1Ai). After centrifuging the adipose tissue, we found that the centrifuged liquid was divided into three layers: the top layer was the digested adipose tissue, the middle layer was the clear solution of type I collagenase, and the bottom layer was a mixed precipitate with mesenchymal stem cells and a small number of erythrocytes (Figure 1Bi). After 48 h of culture, observation under the inverted phase-contrast microscope showed that there were round, nonadherent cells, dead cells, and suspended erythrocytes in the culture medium, and the bottom of the tissue culture vessel had adherent cells, which were spindle-shaped, had few cell protrusions, and were fibroblast-like (Figure 1Ci). After 5 days of culture, there were more cells than at 48 h. However, the cells grew slowly and were long and spindle-shaped, and only few cells were triangular or irregularly shaped (Figure 1Di). Daily observations showed that the cell growth rate gradually accelerated. On the 15^th^ day, the degree of cell confluency was high, and the cell morphology did not change significantly, but the entire population of cells showed a swirling or school-of-fish-like growth trend (Figure 1Ei). Later subcultures showed no obvious morphological or growth changes (Figure 1Fi).

### Immunophenotyping of Adipose Tissue–Derived Mesenchymal Stem Cells

Flow-cytometric immunophenotyping showed that endothelial marker antibody CD31 was negative, the hematopoietic stem cell and endothelial marker antibody CD34 was negative, the leukocyte marker antibody CD45 was negative, the fibroblast marker antibody HLA-DR was negative, and the rest of the mesenchymal stem cell marker antibodies (CD29, CD90, and CD166) were all positive, which can exclude the possibility of other cell types. Flow-cytometric phenotype identification showed that the cells that we isolated and cultured conformed to the immunophenotypic characteristics of Ad-MSCs, that is, the cells obtained from the isolation and culture were Ad-MSCs (Figure 1ii).

## Preparation of Self-Assembled Nanopeptide Hydrogel and Detection by Electron Microscopy

### Preparation of Self-Assembled Nanopeptide Solution

**FIGURE 2:**
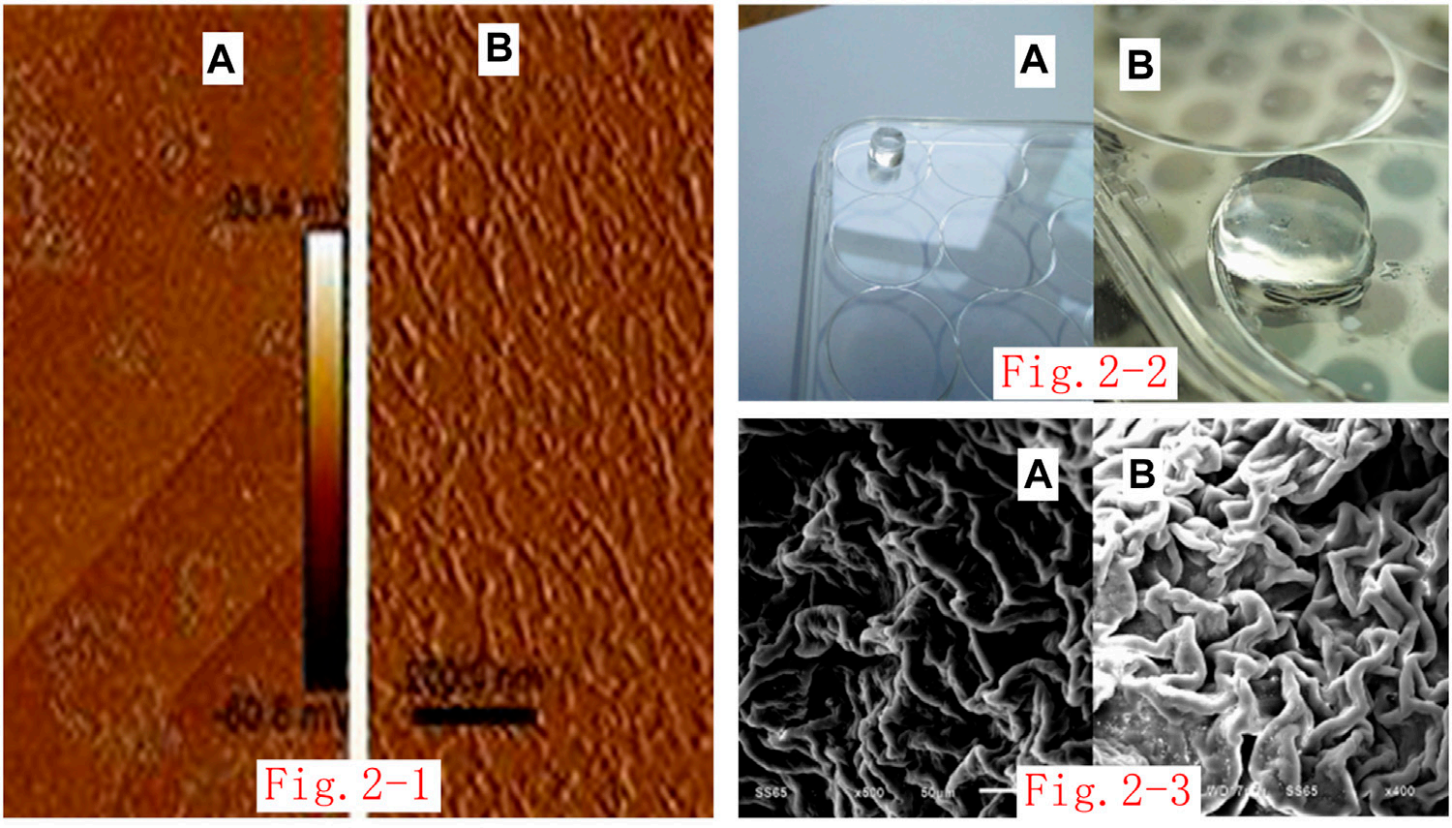
**(i)**. Atomic-force microscope image of self-assembled nanopeptide solution: **(A)**: The nanofiber arrangement can be seen by imaging for the three-peptide mixed solution at 2.0 μm. **(B)**: The nanofiber arrangement can be seen by imaging for the three-peptide mixed solution at 200 nm. **(ii)**. Image of gelation of the nanopeptide solution through the Transwell chamber: **(A)**: Observation of the nanopeptide hydrogel structure from a long distance (the bottom plate is a 12-well plate). **(B)**: Observation of the nanopeptide hydrogel structure from a close distance (the bottom plate is a six-well plate). **(iii)**. SEM observation of the nanopeptide hydrogel: **(A, B)**: SEM observation of the nanopeptide hydrogel shows that its microstructure is tightly arranged, with pores that can accommodate cells (50 μm).

The three self-assembling nanopeptides RADA16-I, RGD, and KLT are all white, powdery substances. When biological-grade, sterile, deionized water was added, the aqueous solution was initially thick and turbid. After an ultrasonic cell pulverizer was used to sonicate the viscous and turbid self-assembling nanopeptide solution, the viscous and turbid peptide solution gradually clarified to become transparent or translucent, indicating that the self-assembling nanopeptide solution had been fully dissolved and mixed. Atomic-force microscope observations showed that the nanopeptides varied in length. They were mostly 200–500 nm in length and 20–50 nm in diameter (Figure 2i).

### Preparation of Self-Assembled Nanopeptide Hydrogel

The self-assembled nanopeptide mixed solution was added to the Transwell chamber and kept in the chamber overnight, and a solid gel structure appeared in the chamber. The basement membrane of the chamber was cut off, and the hydrogel was gently removed. The hydrogel was cylindrical, colorless, and transparent, the texture was loose and brittle, and it was high in water content (Figure 2ii).

### Scanning Electron Microscope Detection of Self-Assembled Nanopeptide Hydrogel

The self-assembled nanopeptide hydrogel was fixed and dehydrated. SEM observation showed that the sample had a fibrous or porous like structure. The diameter of the nanofibers was approximately 20–50 nm, and the cavities were approximately 5–100 nm in size (Figure 2iii).

## Compound Experiments of Self-Assembled Nanopeptide Hydrogel and Adipose Tissue–Derived Mesenchymal Stem Cells

**FIGURE 3:**
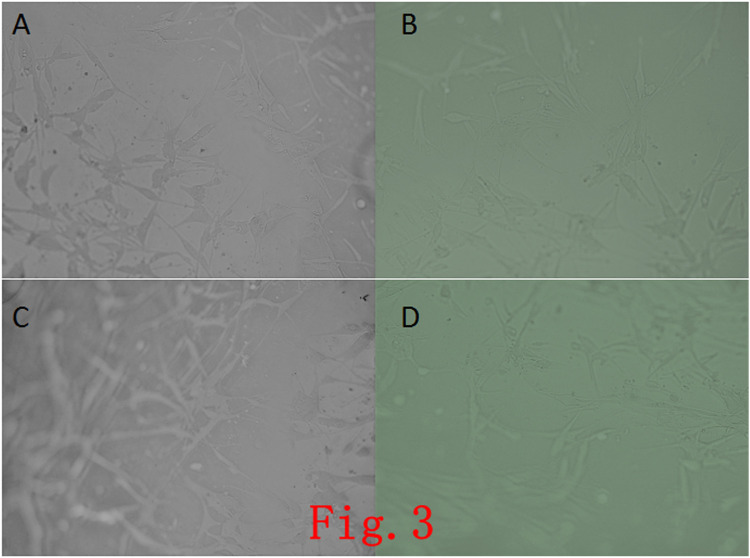
Observation of cells in a self-assembled the nanopeptide hydrogel model combined with Ad-MSCs. **(A)**, **(B)**, **(C)**, **(D)**: Images of cells in the hydrogel photographed at different angles, showing that the cells in the hydrogel have a good growth condition with good adherence (× 100).

Ad-MSCs were inoculated on the self-assembled nanopeptide hydrogel. It was found that the cells were distributed uniformly, and the cell morphology changed slightly, but the cells were in a good state of extension and were tightly connected (Figure 3).

## Self-Assembled Nanopeptide Hydrogels Combined With Adipose Tissue–Derived Mesenchymal Stem Cells as the Biological Ink for 3D-Printed Tissue Models; Induced Differentiation Assay of Cells

### Self-Assembled Nanopeptide Hydrogels Combined With Adipose Tissue–Derived Mesenchymal Stem Cells as the Biological Ink for 3D-Printed Tissue Models

**FIGURE 4:**
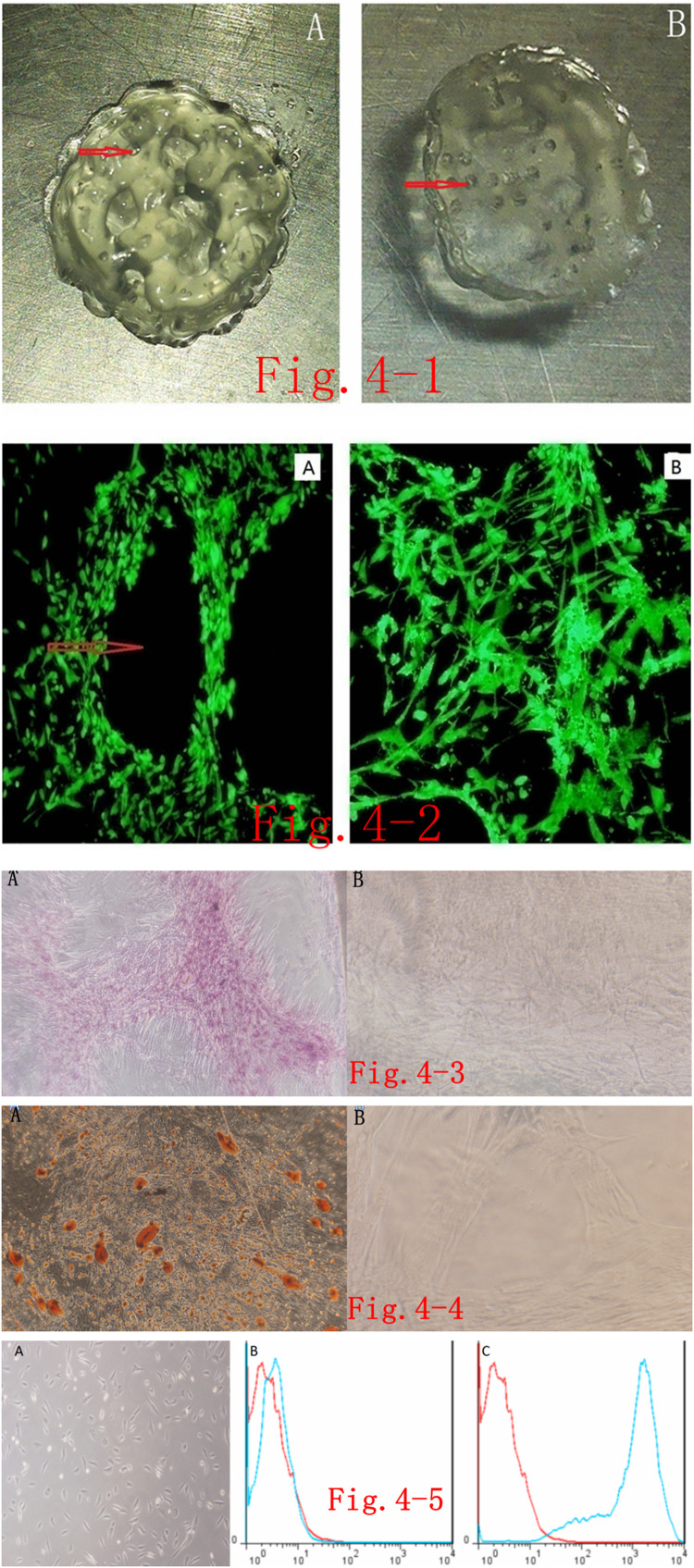
**(i)**. 3D-printed tissue model based on self-assembled nanopeptides and Adipose Tissue–Derived Mesenchymal Stem Cells (Ad-MSCs): **(A)**: Front side of the 3D-printed model, revealing that it is not a regular cylindrical structure and there are pore-like structures as designed by the software. The red arrow in the figure points to the pore (the diameter of the cylinder is 1 cm). On the reverse side of the 3D model, a pore-like structure is still visible (the diameter of the cylinder is 1 cm). **(ii)**. Phalloidin staining of cells in a 3D-printed tissue model: **(A)**: After the 3D-printed model was sectioned and stained, the internal structure was observed by fluorescence microscopy. Red arrows point to pore-like structures (×40). **(B)**: After the 3D-printed model was sectioned and stained, fluorescence microscope observation of the interval structure shows that the cells are arranged in an intricate and tight manner (× 40). **(iii)**. Alizarin red S staining after osteogenic induction of cells in the 3D-printed model: **(A)**: Alizarin red S staining was performed on the Ad-MSCs in the 3D-printed models after osteogenic differentiation and sectioning. Cells were densely packed with red calcium nodule precipitation (× 40). **(B)**: The model without induction of differentiation. Ad-MSCs show no obvious staining (×40). **(iv)**. Oil red O staining of cells in the 3D-printed model after adipogenic differentiation: **(A)**: After adipogenic differentiation of Ad-MSCs in the 3D-printed model, oil red O staining was performed after sectioning. Red lipid droplets are seen in the cells (× 40). **(B)**: Oil red O staining on cells in the models with no adipogenic differentiation. No obvious staining is seen (× 100). **(v)**. Induced endothelial differentiation of cells in the 3D-printed model and immunophenotyping *via* flow cytometry: **(A)**: Induction of endothelial differentiation of Ad-MSCs in the 3D-printed model. Cells demonstrate paving stone–like changes (× 100). **(B)**: CD31-negative expression in the control group. **(C)**: CD31-positive expression in the experimental group.

After mixing the self-assembled nanopeptides with Ad-MSCs and performing 3D printing, the 3D-printed tissue engineering model was a transparent cylinder approximately 1 cm in height and approximately 5 mm in diameter. It had a predesigned porous structure to promote the full contact and material exchange between cells and the culture medium as well as the proliferation, differentiation, and signaling of Ad-MSCs in the 3D-printed tissue model. The materials we used could be printed by a 3D printer, but the printed model was not a regular cylinder in shape, suggesting that the plasticity was slightly poor (Figure 4i).

### Phalloidin Staining of Cells in the 3D-Printed Model

There are many choices of immunofluorescent dyes for immunostaining the printed model. Here, we used phalloidin staining to observe the macroscopic structure of cells in the tissue model, which should have the same effect as other fluorescent dyes. After staining, we found that the cells were in a good growth state, were arranged tightly and orderly, and were in close contact with the model. The porous structures in the model let the cells fully contact the culture medium (Figure 4ii).

### Induced Osteogenic Differentiation of Cells in the 3D-Printed Model and Alizarin Red S Staining

After inducing osteogenic differentiation of cells in the 3D model, sectioning of paraffin-embedded samples, and staining, observation under the microscope revealed that the cells were still closely packed and ordered, and dark-brown calcified nodules appeared in the cells, whereas no obvious staining was seen in the control group. These findings suggest that the induction of osteogenic differentiation and staining in the 3D-printed tissue model were successful (Figure 4iii).

### Induction of Adipogenic Differentiation and Oil Red O Staining of Cells in the 3D-Printed Model

After inducing adipogenic differentiation of cells in the 3D model, sectioning of paraffin-embedded samples, and staining, observation under the microscope revealed that the cells were still arranged tightly and orderly, and there were many lipid droplets in the cells, seen as round, red bubbles, but no obvious staining was seen in the control group. This shows that the induction of adipogenic differentiation and staining in the 3D-printed tissue model was convincing (Figure 4iv).

### Induction of Endothelial Differentiation in 3D-Printed Tissue Models and Flow-Cytometric Identification

After inducing endothelial differentiation of cells in the 3D model, we found that the spindle-shaped cells became shorter, cells presented polygonal or triangular-like changes, and they showed an overall paving stone–like growth, whereas there was no obvious morphological change in the control group (Figure 4v). Flow-cytometric immunophenotyping was performed on the stem cells after induction of endothelial differentiation. After labeling, cells were loaded into the flow cytometer, and it was found that the labeling with endothelium-specific antibody CD31 changed from negative to positive, whereas the control group still had negative CD31 labeling (Figure 4v).

## Discussion

Ad-MSCs have the characteristics of easy culture, strong proliferation ability, and low immunogenicity, offering great possibilities for allogeneic transplantation and an excellent source of seed cells (Zuk et al., 2001). Ad-MSCs also have the most critical ability of stem cells, that is, multidirectional differentiation ability. Under different induction conditions, Ad-MSCs can differentiate into different types of cells, such as osteoblasts, adipocytes, cardiomyocytes, chondrocytes, endothelial cells, and nerve cells. Therefore, Ad-MSCs, as an emerging type of seed cell, have good prospects in the field of regenerative medicine.

In Kar Wey Yong’s study, human fat Mesenchymal stem cell were thawed and subcultured by slow freezing with different cryoprotectants (CPAS) for 3 months, Long term cryopresered hascs maintained normal levels of tumor suppressor markers, and no evidence of DNA damage or p53 mutation was observed, showing that cryopreservation is an effective way to preserve human Mesenchymal stem cell in early culturepassage and to concentrate cells in order to obtain enough ready made cells for clinical use, such as cell-based therapy and regenerative medicine.

In this study, we isolated and cultured a certain number of Ad-MSCs from adipose tissue for passaging and expansion. Observation of the morphological structure revealed that the cells were spindle-shaped or showed fibroblast-like changes, and the entire population showed a swirling or school-of-fish-like growth state. The growth rate of the P0 generation of cells was slow. After passaging, the growth rate of Ad-MSCs gradually increased until it mostly stabilized at P3.

The immunophenotypes for CD29, CD31, CD34, CD45, CD90, CD166, and HLA-DR were typed in this study. CD31 is an endothelial cell–labeling antibody, CD34 is a hematopoietic stem cell– and endothelial cell–labeling antibody, CD45 is a leukocyte-labeling antibody, and HLA-DR is a fibroblast-labeling antibody. Staining using these four antibodies was negative in our study, showing that the isolated and purified cells were not the above cell types. In contrast, CD29, CD90, and CD166 are antibodies labeling Ad-MSCs, and they all showed positive staining in our study, indicating the characteristics of stem cells and showing that our culture had the immunophenotype of Ad-MSCs.

Self-assembled nanopeptides can be transformed from a liquid state to a solid hydrogel structure under specific induction and stimulation conditions, forming many nanofibers (Yong et al., 2017) (Zhang et al., 2005). Self-assembled nanopeptide hydrogels have great advantages in medical research: 1) they are synthesized from natural amino acids under artificial conditions and can be produced in large quantities; 2) these nanopeptides have good biocompatibility, can be degraded into natural amino acids and absorbed by the human body, and have no toxic and side effects; 3) they can be combined with active peptide fragments with different functions to construct new functionalized self-assembled nanopeptide hydrogels (Yuan and Liang, 2013b).

As an extracellular matrix, tissue-engineered materials are of great significance to cell viability (Gelain et al., 2006; Park et al., 2011; Liu et al., 2013). The nanopeptide used in this study was a mixture of three peptides of RADA16-I, RGD, and KLT. After RADA16-I forms a solid-phase hydrogel, the hydrogel contains many pores inside, which is suitable for cell adhesion and migration and beneficial to nutrient transportation. KLT is a vascular growth factor polypeptide fragment that stimulates vascular endothelial growth factor receptors and promotes endothelial cell proliferation (Wang et al., 2014). RGD is a repetitive peptide motif of cell adhesion growth factor and a key binding site for cell adhesion (Ruoslahti, 1996; Hersel et al., 2003) that can promote the adherent growth of cells. Observing the dehydrated self-assembled nanopeptide hydrogel with an SEM revealed that the microstructure in the gel was made of an ordered intertwined porous scaffold structure, the diameter of the nanofibers was approximately 20–50 nm, the pore size was approximately 5–100 nm, and the pores accommodated Ad-MSCs.

Tzu-Wei Wang prepared functional self-assembled nanopeptide scaffolds to promote angiogenesis and neurogenesis in brain injury sites, the scaffold was found to support endothelial cells to form tubular structures and neural stem cells to survive and proliferate (Wang et al., 2017). The results show that the self-assembled Peptide Hydrogel has programmable physical properties, good Biocompatibility and regeneration ability, and can promote the functional recovery of the damaged brain.

We prepared the functionalized self-assembled nanopeptide hydrogel combined with Ad-MSCs for 3D culture. Observation with an inverted phase-contrast microscope showed the same things that SEM of the hydrogel alone did. Ad-MSCs tightly adhered to the polypeptide scaffold and presented a 3D structure. From the above, it is not difficult to see that the functionalized self-assembled peptide hydrogel has the function of an extracellular matrix and can provide cells with a more suitable microscopic environment for survival, promote cell growth, and maintain cell viability.

Alizarin red S is usually used as a staining reagent in the experiments of stem cell osteogenic differentiation. It is a standard method of chemical separation of paraffin and chelating technique, an authoritative and classic technique for the analysis of orange-red calcium deposits in paraffin-embedded tissue sections by producing a complex of calcium ions and Alizarin red S. It is suitable for the detection of calcium deposition and calcium nodules in the study of bone tissue or tissue pathophysiology (Wilson and Boland, 2003).

In the experiment of stem cells into lipogenesis differentiation, oil red O staining is needed. At present, most of the research units use frozen section in fat staining, so they do not undergo any treatment, the less loss of fat particles in the specimen makes the staining more distinct, and the results can be accepted by all.

In the experiment of Stem Cell endothelial differentiation, we used inverted phase contrast microscope to observe the cell morphology in the tissue model. We could find that the spindle cells became shorter and appeared polygonal or triangular shape, in order to further confirm the differentiation of stem cells into endothelial cells, the flow cytometric immunophenotyping of stem cells induced by endothelial differentiation was carried out, the endothelial marker antibody CD31 changed from negative to positive, while the control group remained negative for CD31 Flow cytometry. These two parts of the experiment confirmed the endothelial differentiation of stem cells.

In 2003, Boland et Al introduced the concept of “cell printing,” which is a more complex technology than non-biological printing (Boland et al., 2003). In traditional tissue engineering and regenerative medicine, tissue engineering has been used to treat disease by transplanting scaffolds loaded with pre designed cells, however, there are many limitations: 1) It is difficult to implant different matrix and cells into the scaffold material at the same time to form a model close to the normal structure of the human body; 2) difficulty in accurately accumulating different types of cells and extracellular Matrix in tissue engineering models; 3) scaffold structure itself Limits Cell Migration; 4) scaffold structure itself limits nutrient penetration.

Over the past few decades, printing technology has made the leap from 2D printing to layering and distributing materials to form 3D models. The latest research has made it possible to print living tissue from biocompatible materials, cells and support components using 3D bioprinting, which could be widely used in regenerative medicine for patients who need tissue and organs suitable for transplantation (Huh et al., 2010).

With the development of 3 days bio-printing technology, the limitation of many traditional tissue engineering technologies has been broken. Cell Printing is to print pre-mixed “bio-ink” layer by layer in a 3 days printer to form a 3 days bio-model loaded with cells. The main advantages of 3D cell printing are: 1) the ability to construct a 3D model of multiple cells or extracellular Matrix at the same time; 2) the accurate accumulation of different kinds of cells and different components of biomaterial scaffolds; extracellular Matrix for building cells. Although 3D printing technology has evolved over the years, with some success in printing artificial tissues and organs, these successes have been limited to very thin tissue structures, such as the skin and bladder, but the human body is incredibly complex, 3 days Cell Printing still has a long way to go in tissue or organ construction.

The 3 days printing materials used in our experiment are self-assembled nano-peptide hydrogels, which have many advantages over other materials. We also used the now popular Mesenchymal stem cell, which is widely used in tissue engineering because of its seed cell specific advantages, accessibility, and low immunogenicity. In this study, we used 3D printing, self assembling Nanopeptides, and fat Mesenchymal stem cell. Self assembled nanopeptides support and support cells as extracellular scaffolds, while functional self assembled nanopeptides have extracellular matrix effects. As the seed cell of this printing, the Mesenchymal stem cell has many advantages, providing the basic conditions for printing biologically active tissue (Sonntag et al., 2010).

The “bio-ink” is prepared by processing the self-assembled nano-peptide mixture with the FAT Mesenchymal Stem Cell for printing. Then, the bio-ink was added to the sterile bio-ink cartridge by bio-printer, and printed layer by layer on PBS substrate. The 3 days model was approximately cylindrical in diameter and 0.5 cm in height, it has a number of small holes in it, which is important for the cell to exchange material with its surroundings, so the material we use can be printed on a 3 days printer, but the printed model is not a regular cylinder, so it’s a little less malleable. The culture of the cells in the printed tissue model is similar to the three-dimensional culture of the cell model, and the operation of changing the solution is similar to that of the culture bottle, but it is necessary to use the complete medium to clean the model thoroughly several times when changing the solution, to clean the remaining medium in the small hole of the model. After paraffin sectioning and Phalloidin staining, the cells were arranged in a compact and orderly manner, the cells were in a good state of growth and adhered closely to the wall, and the hole-like structure left by the printing process could be seen under the Fluorescence microscope, it shows that the cells can grow normally after 3D printing. The cells in the printed tissue model were induced to differentiate and stained by the corresponding induction conditions, the staining of the cells showed that the cells differentiated after 3D printing in the tissue model under the corresponding induction conditions (Günther et al., 2010).

We used Ad-MSCs as seed cells in this study because they have many advantages, such as easy availability and low immunogenicity. Self-assembled nanopeptides are used to support cell growth and play the role of extracellular matrix. Ad-MSCs in the 3D-printed tissue models using a mixed solution of self-assembled nanopeptides and Ad-MSCs as the biological ink showed good growth status and still had strong differentiation ability.

## Data Availability

The raw data supporting the conclusions of this article will be made available by the authors, without undue reservation.

## Abbreviations

Ad-MSCs, adipose-derived mesenchymal stem cells; DMEM, dulbecco’s modified Eagle’s medium; EDTA, ethylene diamine tetraacetic acid; FBS, fetal bovine serum; HLA, human leukocyte antigen; PBS, phosphate-buffered saline; SEM, scanning electron microscope.
